# 

**Published:** 1898-07-09

**Authors:** 


**Notices of Births, Deaths, and Marriages** are inserted in this column at a charge of 2s. 6d. for an announcement not exceeding four lines.

### NOTICE TO CORRESPONDENTS.

All MS., Letters, Books for Review, and other matters intended for the Editor should be addressed The Editor, "The Hospital," The Hospital Building, 28 & 29, Southampton Street, Strand, W.C.

The Editor cannot undertake to return rejected MS., even when accompanied by stamped directed envelope.

**Notice to Advertisers and Subscribers.**

All Advertisements, Orders for Copies of The Hospital, and Business Communications should be addressed to **THE MANAGER, (Not to The Editor),** The Hospital, The Hospital Building, 28 & 29, Southampton Street, Strand, London, W.C.

The Cost of Subscription, post free, is as follows:—Per week, 2½d. per quarter, 2s. 8d.; per half-year, 5s. 3d.; per annum, 10s. 6d. Foreign:—Per annum, 15s. 2d.; payable, in all cases, in advance.

**NOTICE.—Vol. XXIII. of "The Hospital" (October, 1897, to March, 1898), in handsome cloth binding, is now on sale at the Office of this Journal, price 7s. 6d. Cases for binding the Numbers contained in this Volume, 1s. 6d. Index to Vol. XXIII., 2½d., post free.**

**The Monthly Part for July is now ready. Price 6d., by post 8d.**

### BOOKS RECEIVED.

T. Fisher Unwin.

"Sir Benjamin Collins Brodie." By Timothy Holmes, M.A., F.R.C.S. Masters of Medicine Series.

Simpkin, Marshall, Hamilton, Kent, and Co.

"Copenhagen and its Environs."

Religious Tract Society.

"Health at Home." By A. T. Schofield, M.D.

Bemrose and Sons.

"The People's Guide to the Workmen's Compensation Act."

Smith, Elder, and Co.

"The Mineral Waters and Health Resorts of Europe." By Hermann Weber, M.D., F.R.C.P., and F. Parkes Weber, M.D., F.R.C.P.

William F. Clay, Edinburgh.

"Guide to the Examination and Treatment of Sick Children." By John Thomson, M.D., F.R.C.P.Ed.

Bailliere, Tindall, and Cox.

"Cancerous and Other Tumours." By Herbert Snow, M.D.Lond.

"A Manual for Students of Massage." By M. A. Ellison, L.O.S.

Whittaker and Co.

"The Chemist's Compendium." By C. J. S. Thompson.

The Great Eastern Railway.

"Tourist Guide to the Continent." Edited by Percy Lindley.

